# Bridging riverine and lacustrine systems: Macroinvertebrate indicators of ecological health in the Rwandan Congo basin

**DOI:** 10.1007/s10661-025-14641-y

**Published:** 2025-10-20

**Authors:** Gilbert Ndatimana, Marie Claire Dusabe, Christian Albrecht

**Affiliations:** 1 Department of Animal Ecology & Systematics, Justus Liebig University Giessen, Heinrich-Buff-Ring 26 (IFZ), 35392 Giessen, Germany; 2 Department of Biology, Mbarara University of Science and Technology, Mbarara, Uganda; 3 Center for Environmental Research and International Development (ZEU), Justus Liebig University Giessen, Senckenbergstrasse 3, 35390 Giessen, Germany

**Keywords:** Ecological Quality Ratio (EQR), Multimetric index (MMI), River Continuum Concept (RCC), Lake Kivu, Biomonitoring

## Abstract

Connectivity between lakes and tributaries is essential for freshwater ecosystem health. However, integrated studies of macroinvertebrate assemblages across these interconnected systems remain limited in tropical Africa. This study examined the structure of the macroinvertebrate community and the ecological health of Lake Kivu and its tributaries using multimetric indices. Macroinvertebrates were collected from 23 lake sites and 53 tributary sites between 2010 and 2022. Diversity indices were quantified, community differences were tested using permutational multivariate analysis (PERMANOVA), whereas Indicator value analysis (Indval) was performed to identify ecosystem-taxa associations. The ecological health status of the lake was assessed using the Multimetric Index for Lake Hawassa (MMIH), and the Macroinvertebrate-Based Multimetric Index for Biotic Integrity (MMIBI) for tributaries. Lake and tributary systems exhibited distinct community compositions, with molluscs dominating the lake and insects prevalent in tributaries. Families Thiaridae, Bithyniidae and Planorbidae were prevalent in the lake, while Baetidae and Chironomidae were common in the tributaries. Indicator species analyses revealed strong habitat affinities of *Melanoides tuberculata* and *Gabbiella humerosa* to the lake, and *Radix natalensis* and *Pisidium kenianum* to the rivers. The diversity indices indicated significantly higher macroinvertebrate diversity in the tributaries (*p* < 0.001), whereas molluscan diversity was greater in the lake (*p* < 0.05). The results classified the lake sites as having “fair” to “good” ecological status, while the tributaries ranged from “poor” to “moderate”, reflecting the varying levels of anthropogenic pressure. These findings highlight the ecological distinctiveness of lacustrine and riverine systems, and demonstrate the effectiveness of using macroinvertebrates for integrated, basin-scale monitoring and management.

**Supplementary information:**

The online version contains supplementary material available at 10.1007/s10661-025-14641-y.

## Introduction

The connection between a lake and its catchment is important in shaping the lake characteristics and its responses to catchment changes (Anderson, 2014; Vilbaste et al., 2024). Lakes connect to their catchment mainly by direct inflow through rivers and surface runoff. The catchment influence lakes and rivers differently due to various factors such as topography, human activities and the nature of the riparian zone (Dai et al., 2017). As rivers meander through the catchment, they collect organic and mineral materials, which are washed into the lake. This eventually affects the chemical and ecological dynamics of the receiving ecosystem (Kling et al., 2000; Jones 2010). In fact, systems connecting through agricultural, urbanized and industrialized areas are affected by organic and inorganic pollution (Lei, 2010; Ozdemir et al., 2025). Further, major infrastructures such as dams, diversion for hydropower production, irrigation and municipal water supply significantly deplete water volume in both the rivers and the receiving lake (Unnikrishnan et al., 2021). The fluctuations in water volume affect water quality through changes in temperature, dissolved oxygen, and nutrient concentrations, ultimately affecting habitats stability (Mosley et al., 2012; Zerlin & Henry, 2014). Consequently, lake biodiversity often declines abruptly due to habitat destruction, deteriorating water quality, and increased exposure to extreme environmental conditions in the catchment (Akbayeva et al., 2025; Panigrahi, 2007). However, the extent of these impacts depends on the characteristics of both the tributaries and the receiving lake, such as shape and size (Jones 2010).

Riverine biodiversity is shaped by the structure and composition of riparian zones (Masese et al., 2014a). It also varies along a longitudinal gradient described by the River Continuum Concept (RCC), which integrates biotic and abiotic factors from headwater to mouth (Vannote et al., 1980). Key abiotic variable such as sediment composition, temperature, light availability, flow regime, and water chemistry play key roles in shaping community assemblages (Bækkelie et al., 2017; Shuman et al., 2020). Macroinvertebrate communities shift in structure along the river continuum in response to changes in resource availability and habitat conditions, reflecting the RCC’s framework of ecological integration (Benjamin et al., 2025). At the river–lake interface, a distinct ecotone forms where riverine and lacustrine systems interact, supporting higher macroinvertebrate diversity than found in lakes alone (Hillbricht-Ilkowska & Węgleńska, 2003). This zone is shaped by flow dynamics and hydro-morphological conditions (Jones 2010; Pennock et al., 2021), as the mixing of chemically and physically distinct waters promotes organic matter deposition and reduces flow velocity (Tian et al., 2020). Here, the convergence of upstream allochthonous inputs and lake backflows further enhances nutrient retention, biomass, and overall ecological productivity (Lin & Guo, 2016; Tian et al., 2020). Due to their ecological sensitivity and well-defined traits, macroinvertebrates are widely used as indicators in assessing the condition of riverine and lacustrine ecosystems (Assie et al., 2025; Masese et al., 2014a).

Several approaches have employed macroinvertebrates as bioindicators to evaluate ecological health of riverine and lacustrine systems (Brown & Williams, 2016; Fierro et al., 2017; Kaaya et al., 2015; Koperski, 2011; Ndatimana et al., 2023a). In Africa, biomonitoring using macroinvertebrate-based indices are biased in riverine ecosystems (Ndatimana et al., 2023b; Odountan et al., 2019). Traditionally, taxa presence/absence and diversity indices such as SASS (Dickens & Graham, 2002) and TARISS (Kaaya et al., 2015) were developed to guide the assessment of rivers in African regions. These Sensitivity-based indices provide insights into the significance of macroinvertebrates in ecosystem monitoring and conservation (Tampo et al., 2021). Combining various metrics, including traditional ecological indices (e.g., alpha diversity), macroinvertebrate traits such as functional feeding groups (FFGs), and composition, multimetric indices (MMIs) were developed (e.g., Alemneh et al., (2019); Kaboré et al., (2022); Wondmagegn and Mengistou, (2023) and Ndatimana et al., (2023a). This approach accounts for various aspects of habitat and reduces errors in sensitivity scores of certain taxa (Chen et al., 2019). In African regions where there are no specific indices, water quality assessment relies on indices from ecologically similar areas, as well as generalized sensitivity-based indices. For instance, the application of macroinvertebrate-based assessments in Rwanda have employed the TARISS (e.g., Dusabe et al., 2019, and Nsengimana et al., 2025), alongside generalized indices such as ASPT, The Baur biotic Score, BMWP and other traditional ecological indices (e.g.: Wronski et al., 2015).

Lake Kivu, Rwanda’s largest water body (Nsabimana et al., 2020; Olapade & Omitoyin, 2012), has garnered considerable attention from researchers for various reasons. Its deep layers contain dissolved carbon dioxide (CO_2_) and methane (CH_4_), making it potentially susceptible to explosive gas release (Hirslund & Morkel, 2020). Moreover, besides being deep (Ross et al., 2014) and at high altitude (Degens et al., 1973), it was also claimed to have relatively low diversity, compared to other African great lakes (Dusabe et al., 2024; Sarmento et al., 2007). Lake Kivu is prone to pollution and disturbances due to land use and land cover changes in its catchment (Bagalwa et al., 2015; Hahirwabasenga et al., 2024; Olapade & Omitoyin, 2012). The urbanization increases around the lake basin with towns like Rubavu, Karongi and Rusizi in Rwanda (Bundervoet et al., 2017; Turatsinze et al., 2024), and Goma and Bukavu in DR Congo (Bayumbasire et al., 2021) with a population of 5.7 million people (Muvundja et al., 2023). The increasing agricultural activities in the basin contribute to nutrient loading into the lake from used fertilizer and pesticides, which are carried through rivers (Bagalwa et al., 2015; Muvundja et al., 2009). This compromises water quality and survival of inhabiting organisms. As the risk of pollution increases, the growing need for water quality assessment is needed to safeguard the ecological integrity of the ecosystem. Indeed, on the Rwandan side of Lake Kivu basin, water pollution assessed using physico-chemical indices was reported in some parts of the lake and its tributaries (Mupenzi et al., 2017; Nsabimana et al., 2020). However, studies on the application of macroinvertebrates in assessing the water quality of the Kivu basin are still limited. The available studies in this region focused on rivers or the lake separately (Dusabe et al., 2024; Hyangya et al., 2022; Wronski et al., 2015), with minimal attention to their connectivity. Consequently, there is limited information on macroinvertebrate assemblages of Lake Kivu and its tributaries, their associated water quality, and the overall ecological health of these interconnected yet distinct systems within the basin.

This study aimed at assessing the ecological integrity of Rwandan part of the Congo Basin using macroinvertebrates, specifically:

1. Comparing the riverine macroinvertebrate community structure to that in Lake Kivu.
2. Testing the ecological health status of Lake Kivu and its tributaries, by applying the selected macroinvertebrate-based MMIs available in the region.

Understanding macroinvertebrate patterns as bioindicators in the lake and its tributaries is vital for assessing the interactions of interconnected systems, supporting effective water resource management and conservation in the basin.

## Methods

### Study area

**Fig. 1:**
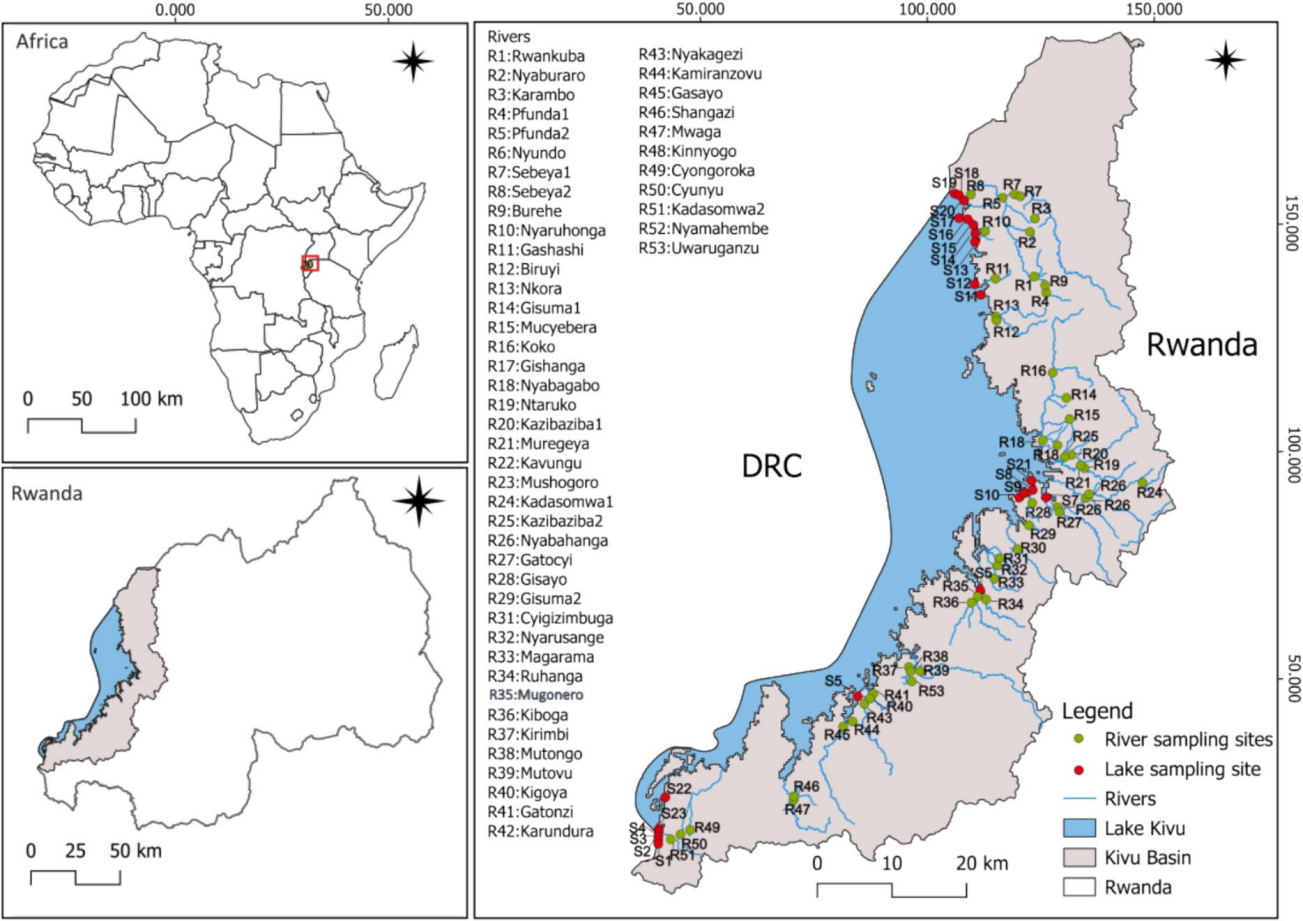
Map showing the study sites within the Rwandan part of the Congo Basin, including sampling points for Lake Kivu and its tributaries (shown on the right). Insets of the map of Africa (top left) and Rwanda (bottom left) are also presented for geographic context

The study was carried out in the Lake Kivu drainage system in Rwanda, including both the lake and its tributaries (rivers) (Fig. 1). Lake Kivu forms part of the western branch of the East African rift valley system (Dusabe et al., 2024; Ross et al., 2014). It is situated at an elevation of 1,463 m above sea level (Degens et al., 1973) and is shared between Rwanda and Democratic Republic of the Congo with a catchment area of 5097 km^2^ (Muvundja et al., 2023). It has a surface area, volume and a maximum depth of 2,370 km^2^, 549 km^3^ and 486 m, respectively (Muvundja et al., 2023; Ross et al., 2014). The region receives an average annual precipitation of 1,404 mm, and is characterized by a humid climate with a bimodal precipitation pattern. The rainy season extends from September to May, while the dry season occurs from June to August. The rainy season is further divided into a long rainy period from February to May and a short rainy period from October to December (Muvundja et al., 2014; Dusabe et al., 2024). Precipitation is the primary contributor to the lake’s water budget, accounting for 49% of the total inflow. Riverine input from more than 100 small tributaries constitutes 32%, while sub-aquatic springs contribute the remaining 20% (Muvundja et al., 2014; Muvundja et al., 2023). Lake Kivu has two primary outflow pathways; Rusizi River, accounts for approximately 54% of the total outflow, and evaporation which accounts for the remaining 46% (Muvundja et al., 2014).

The Rwandan part of Kivu basin, cover 33% of Rwanda’s total area and receive 10% of the total national water through various rivers (REMA, 2009; Mupenzi et al., 2017). The lake catchment through which its rivers meanders mainly in hills with some wetlands and dominated by agricultural activities. Moreover, the Industrial activities such as breweries and other agri-based processing, are particularly done in the cities adjacent to the lake (Wronski et al., 2015). Land cover in the catchment comprises 65% dryland, cropland, and pasture, 17% evergreen broadleaf forest, 11% shrubs, and 7% other land types (Muvundja et al., 2009). Over time, much of the 17% evergreen forests, which buffered numerous rivers examined in this study, have been gradually replaced by farmland, pasture, and exotic tree species like Australian Acacia and Eucalyptus (Wronski et al., 2015). In the increase rate of deforestation coupled with steep terrain of the catchment area has increased the risk of soil erosion and landslides (Karamage et al., 2016).

### Macroinvertebrates sampling

Macroinvertebrates were collected from Lake Kivu between October and November 2022, covering the northern, middle, and southern parts of the Rwandan catchment. Further, the study incorporated samples previously collected in 2010, 2018, and 2019. Samples were collected from 23 points, of which ten, six and seven points were from the northern, middle and southern part, respectively (Fig. 1, Table 1). Macroinvertebrates were sampled from the littoral zones of the lake using a 20 cm diameter scoop net (1 mm mesh), and an Ekman grab to collect specimen from soft sediment, while a Surber net with 250 µm mesh size was employed to sample hard substrates. Meanwhile, 53 tributaries from 29 catchments sampled by Wronski et al., 2015 were reused in this study, covering 13, 23, and 17 rivers from northern, middle and southern part respectively (Fig. 1, Table 2). Each river was sampled once between December 2013 to May 2014. The selection of the sampling points was based on river habitats and substrates (Wronski et al., 2015). Macroinvertebrate collection was done using two methods, kick sampling and hand net sampling. After rinsing, the collected macroinvertebrates were pooled together, sorted, and placed into plastic containers.

**Table 1:** The list and location of the sampled rivers with their respective basin section

S/N	Latitude	Longitude	Altitude	Date	River	Catchment
Northern section						
R1	S01.80151	E029.35048	2028	Feb 2014	Rwankuba	Sebeya
R2	S01.74616	E029.34263	2047	Feb 2014	Nyaburaro	Sebeya
R3	S01.73303	E029.34458	1989	Feb 2013	Karambo	Sebeya
R4	S01.82026	E029.35886	2072	Dec 2013	Pfunda1	Sebeya
R5	S01.70939	E029.30865	1683	Dec 1013	Pfunda2	Sebeya
R6	S01.70724	E029.32755	1814	Dec 2013	Nyundo	Sebeya
R7	S01.70251	E029.32708	1795	Dec 1013	Sebeya1	Sebeya
R8	S01.70494	E029.26190	1472	Feb 2014	Sebeya2	Sebeya
R9	S01.81956	E029.35815	2096	Dec 2013	Burehe	Burehe
R10	S01.74711	E029.28240	1434	Dec 2013	Nyaruhonga	Nyaruhonga
R11	S01.80420	E029.29734	1606	Feb 2014	Gashashi	Gashashi
R12	S01.85009	E029.29918	1470	Feb 2014	Biruyi	Nkora
R13	S01.85009	E029.29918	1470	Feb 2014	Nkora	Nkora
Middle section						
R14	S01.95159	E029.38412	1910	April 2014	Gisuma1	Koko
R15	S01.97529	E029.39048	1994	April 2014	Mucyebera	Koko
R16	S01.91596	E029.36395	1629	April 2014	Koko	Koko
R17	S02.00057	E029.35705	1434	April 2014	Gishanga	Gishanga
R18	S02.00250	E029.37395	1622	April 2014	Nyabagabo	Nyabagabo
R19	S02.03045	E029.39901	1527	April 2014	Ntaruko	Muregeya
R20	S02.02013	E029.39095	1631	Feb 2014	Kazibaziba1	Muregeya
R21	S02.02994	E029.39874	1529	April 2014	Muregeya	Muregeya
R22	S02.06699	E029.41105	1581	April 2014	Kavungu	Mushogoro
R23	S02.06406	E029.41335	1582	April 2014	Mushogoro	Mushogoro
R24	S02.05043	E029.47610	2136	April 2014	Kadasomwa1	Mushogoro
R25	S02.01986	E029.39097	1625	Feb. 2014	Kazibaziba2	Muregeya
R26	S02.08145	E029.37558	1478	April 2014	Nyabahanga	Nyabahanga
R27	S02.08257	E029.37512	1479	April 2014	Gatocyi	Nyabahanga
R28	S02.07200	E029.34411	1490	April 2014	Gisayo	Gisayo
R29	S02.10213	E029.33362	1467	April 2014	Gisuma2	Kamaramaka
R30	S02.13135	E029.32529	1561	April 2014	Kiraro	Kiraro
R31	S02.14219	E029.30565	1562	April 2014	Cyigizimbuga	Cyigizimbuga
R32	S02.14613	E029.30117	1553	April.2014	Nyarusange	Nyarusange
R33	S02.16687	E029.29632	1533	April 2014	Magarama	Magarama
R34	S02.19228	E029.28585	1470	April 2014	Ruhanga	Ruhanga
R35	S02.19117	E029.27752	1463	April 2014	Mugonerp	Mugonero
R36	S02.19349	E029.27254	1476	April.2014	Kiboga	Kiboga
Southern section						
R37	S02.27201	E029.19338	1482	May 2014	Kirimbi	Kirimbi
R38	S02.28011	E029.19550	1479	May 2014	Mutongo	Kirimbi
R39	S02.28086	E029.20691	1486	May 2014	Mutovu	Kirimbi
R40	S02.30781	E029.15141	1478	May 2014	Kigoya	Kigoya
R41	S02.30724	E029.15289	1479	May 2014	Gatonzi	Kigoya
R42	S02.31111	E.029.14303	1474	May 2014	Karundura	Karundura
R43	S02.31135	E029.14361	1471	May 2014	Nyakagezi	Karundura
R44	S02.34159	E029.11832	1463	May 2014	Kamiranzovu	Kamiranzovu
R45	S02.34417	E029.09553	1510	May 2014	Gasayo	Gasayo
R46	S02.43086	E029.05773	1527	May 2014	Shangazi	Cyongoroka
R47	S02.43086	E029.05773	1527	May 2014	Mwaga	Cyongoroka
R48	S02.446.83	E028.96992	1675	May 2014	Kinnyogo	Gisuma
R49	S02.47096	E028.93147	1649	May 2014	Cyongoroka	Cyunyu
R50	S02.47425	E028.92357	1627	May 2014	Cyunyu	Cyunyu
R51	S02.48265	E028.90944	1574	May 2014	Kadasomwa 2	Kadasomwa
R52	S02.444.03	E028.97657	1659	May 2014	Nyamahembe	Gisuma
R53	S02.28043	E029.19753	1482	May 2014	Uwaruganzu	Kirimbi

*R* stands for river

**Table 2:** The location of sampling sites on the lake with their respective basin sections

S/N	Latitude	Longitude	Date
Northern section			
S11	−1.81336	29.2795	October 2022
S12	−1.813	29.27903	October 2022
S13	−1.74814	29.27814	October 2022
S14	−1.74014	29.27461	November 2022
S15	−1.74006	29.27511	November 2022
S16	−1.73939	29.27028	November 2022
S17	−1.73847	29.27542	November 2022
S18	−1.70307	29.25801	June 2010
S19	−1.69798	29.24905	May 2019
S20	−1.70423	29.25916	June 2010
Middle section			
S6	−2.18839	29.28014	November 2022
S7	−2.07361	29.35914	November 2022
S8	−2.06222	29.34678	November 2022
S9	−2.06086	29.34578	November 2022
S10	−2.06036	29.33725	November 2022
S21	−2.05329	29.34718	June 2010
Southern section			
S1	−2.48436	28.89408	November 2022
S2	−2.48408	28.89364	November 2022
S3	−2.48322	28.89428	November 2022
S4	−2.47922	28.89847	November 2022
S5	−2.31239	29.13775	November 2022
S22	−2.43384	28.90397	June 2010
S23	−2.4753	28.89877	June 2010

All macroinvertebrates’ samples (from both lake and rivers) were preserved in 70% Ethanol and transported to the University of Giessen Systematics and Biodiversity collection (UGSB), Giessen for further processing and identification. Due to lack of detailed macroinvertebrate identification guides for genera and species in the region, the identification was mainly done to family level using keys by Day et al. (1999, 2001a & b, 2002); Day and de Moor (2002); de Moor and Day (2002); de Moor et al. (2003); Stals and de Moor (2007). However, molluscs were identified to the species level whenever possible using identification key by Brown (1994). In cases where a shell-based morphological approach limited identification to the species level, DNA barcoding was used to improve taxonomic resolution. The tissue samples were used for DNA extraction, the standard barcoding fragment of the COI gene was amplified, sequenced, then the resulting sequences were compared with reference GenBank to confirm species identity.

## Data analysis

### Macroinvertebrate community structure

Macroinvertebrate community patterns and characteristics were investigated across the region (lake and tributaries). All analyses were performed using R software (R Core Team, 2024). Clustered heatmaps (pheatmap package; Kolde, 2019) were generated to visualize similarities among sites and macroinvertebrate taxa for both rivers and lakes. This was done to understand the similarities among sampling stations for both rivers and lakes. To handle skewness, the raw data (abundance) were log (x + 1) transformed for further analysis (Heino, 2008). The difference in assemblage composition between the lake and its tributaries, were performed using the permutational multivariate analysis of variance (PERMANOVA) based on Bray–Curtis distance matrix through “adonis2” function of Vegan package, with 999 Permutations (Oksanen et al., 2022). A similar analysis was applied to the molluscan dataset (identified at species level).

Indicator species analysis (Indval) was applied to different taxonomic resolutions. The overall data were assessed at the family level, whereas molluscan species were analyzed at the species levels to identify the taxa distinguishing the lake from the tributaries. This analysis evaluates the association of taxa (in this case, either families or species) with sample groups (here defined as water bodies, including Lake Kivu and tributary assemblages) by analyzing occurrence data and the relative abundances of individual taxa. Based on the results, the algorithm calculates indicator values (Indval) statistics, which range from 0 to 1 for non-indicator to the best indicator taxa respectively, along with their associated probabilities. The indicator analysis was conducted using the “indicspecies” package (Cáceres & Legendre, 2009), with 999 permutations and a significance level of α = 0.05. The Indicator Value (IndVal) method identifies species that are strongly associated with particular habitats based on their frequency and exclusivity (Dufrêne & Legendre, 1997). The assessment of diversity, four major indices were selected to inform diversity patterns of macroinvertebrates. Shannon–Wiener index, Simpson (dominance), Margalef (richness), and Pielou (evenness) were computed. To investigate the diversity variation between lake and river, the diversity indices were tested and visualized using two-tailed Wilcoxon test and box plots respectively. The biodiversity indices were computed and visualized using packages “Vegan” and “tidyverse” (Oksanen et al., 2022; Wickham et al., 2019).

### Ecological health status of water bodies

The study area was divided into three sections: the north (13 rivers, 10 lake sites), the center (23 rivers, 6 sites), and the south (17 rivers, 7 sites) (Table 1) to assess ecological variability across the basin. River and site selection was based on accessibility, habitat representativeness and historical monitoring data. The ecological health status of the lake and rivers was assessed through the application of macroinvertebrate-based indices available in the region. Macroinvertebrate-based approaches suitable for assessing and comparing the water bodies in the basin were critically selected. Although presence-absence indices are developed and applied for rivers in the region (Dickens & Graham, 2002; Kaaya et al., 2015), they were excluded in the analysis, since no such index is documented for lakes. To ensure effective comparison between lake and its tributaries, multimetric indices (MMIs) developed in the region were used. Indeed, MMIs factors in aspects beyond presence-absence, such as diversity, composition, and traits (Alemneh et al., 2019; Chen et al., 2019). The multimetric index for Lake Hawassa (MMIH), Ethiopia (Wondmagegn & Mengistou, 2023), and macroinvertebrate-based multimetric index for biotic integrity (MMIBI) for Lake Victoria tributaries, Kenya (Aura et al., 2021) were applied for the lake and rivers, respectively. During the application, 10 metrics for MMIH were computed based on the lake dataset, while 11 metrics for MMIBI were computed on rivers (Aura et al., 2021). Ecological Quality Ratio (EQR) was calculated by dividing the observed ecological quality value for each habitat by respective reference conditions. This enabled comparison of the water quality between the lake and rivers. Indeed, EQR is essential for standardizing and calibrating the assessment of ecological status, ensuring effective comparison across various index scales (Bennett et al., 2011; Navarro et al., 2009).

## Results

### Macroinvertebrate composition and diversity

**Fig. 2:**
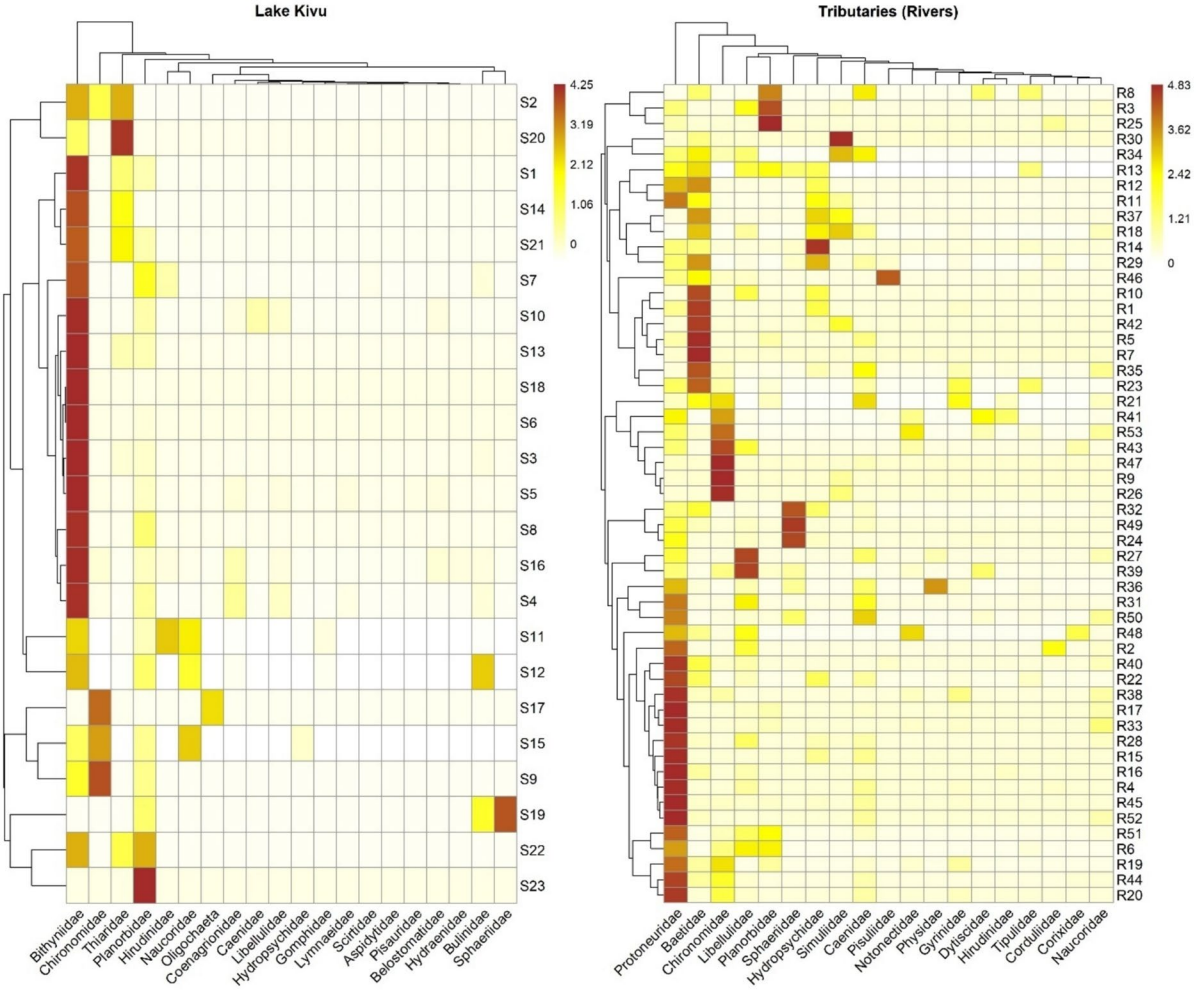
Heatmaps showing the distribution and relative abundance of macroinvertebrate families across sampling sites in Lake Kivu and tributary rivers. Color intensity indicates relative abundance, with hierarchical clustering applied to highlight ecological similarity

A total of 2362 individuals from 16 orders and 27 families were collected from Lake Kivu (Appendix 1). Littorinimorpha, Hygrophila, and Odonata orders dominated with 68.33%, 12.61%, and 4.57%, respectively of the total abundance. The most represented families in lake were Bithyniidae (68.33%) and Planorbidae (9.1%), followed by Hirudinidae (3.38%) and Naucoridae (3.09%) (Appendix 1). Among molluscs, 1894 individuals belonging to nine species were recorded in the lake, with *Gabbiella humerosa* being the dominant species (85.21%), followed by *Biomphalaria pfeifferi* (8.23%) (Appendix 3). On the other hand, 4204 individuals (17 orders and 48 families) were collected from rivers (Appendix 2). Odonata (36.13%), Ephemeroptera (16.15%), Hygrophila (13.79%), and Diptera (13.77%) were the dominant orders while Baetidae (10.94%), Chironomidae (9.06%), Lymnaeidae (8.18%), and Sphaeriidae (7.20%) dominated the families (Fig. 2). Moreover, 878 molluscan individuals recorded in rivers were represented by four species i.e., *Radix natalensis* (39.06%), and *Pisidium kenianum* (34.51%), *Biomphalaria pfeifferi* (21.41%) and *Physella acuta* (5%) (Appendix 4).

Lake macroinvertebrate composition differs significantly from that of tributaries (PERMANOVA, *p* < 0.001, Perms = 999). Further, the species composition of molluscs was different in the two habitats (PERMANOVA, *p* < 0.001, Perms = 999). The indval analysis (significance, *p* < 0.05) showed 5 families with a strong affinity to the lake, including Thiaridae (*p* = 0.001), Coenagrionidae (*p* = 0.002), Bulinidae (*p* = 0.01), Atyidae (*p* = 0.004), and Gomphidae (*p* = 0.039). Further, three species, *Melanoides tuberculata* (*p* = 0.001), *Afrogyrorbis kigeziensis* (*p* = 0.001), and *Gabbiella humerosa* (*p* = 0.001) were found to be significantly associated with the lake, indicating their strong affinity for its environmental conditions. Meanwhile, the rivers were significantly associated with families Protoneuridae (*p* = 001), Gyrinidae (*p* = 0.004), Caenidae (*p* = 0.005), Hydropsychidae (*p* = 0.01), Libellulidae (*p* = 0.022), and Baetidae (*p* = 0.029). Specifically, two molluscan species; *Radix natalensis* (*p* = 0.001) and *Pisidium kenianum* (*p* = 0.003) were associated with the tributaries, with the latter occurring exclusively in these systems.

**Fig. 3:**
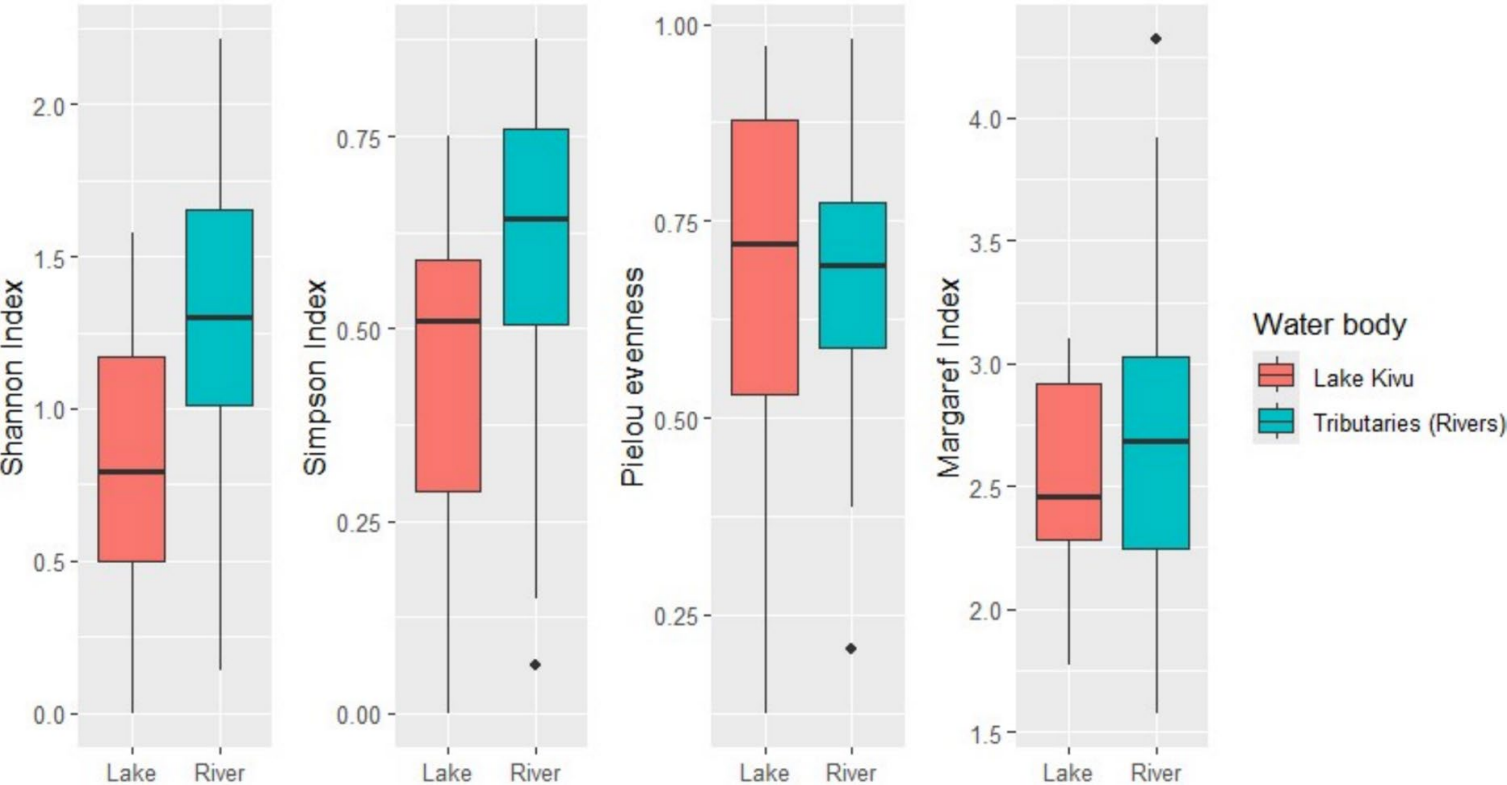
Diversity indices of macroinvertebrate families from Lake Kivu (*N* = 2363) and its tributaries (*N* = 4204)

With reference to diversity indices (Shannon, Simpson, Pielou, and Margalef), there were higher taxa diversity and dominance in tributaries than the lake as indicated by Shannon index (*p* < 0.001), and Simpson index (*p* = 0013), respectively (Fig. 3). However, there was no significant difference in taxa richness and evenness between the lake and tributaries based on Margaref index (*p* = 0.46) and Pielou evenness (*p* = 0.52) (Fig. 3).

**Fig. 4:**
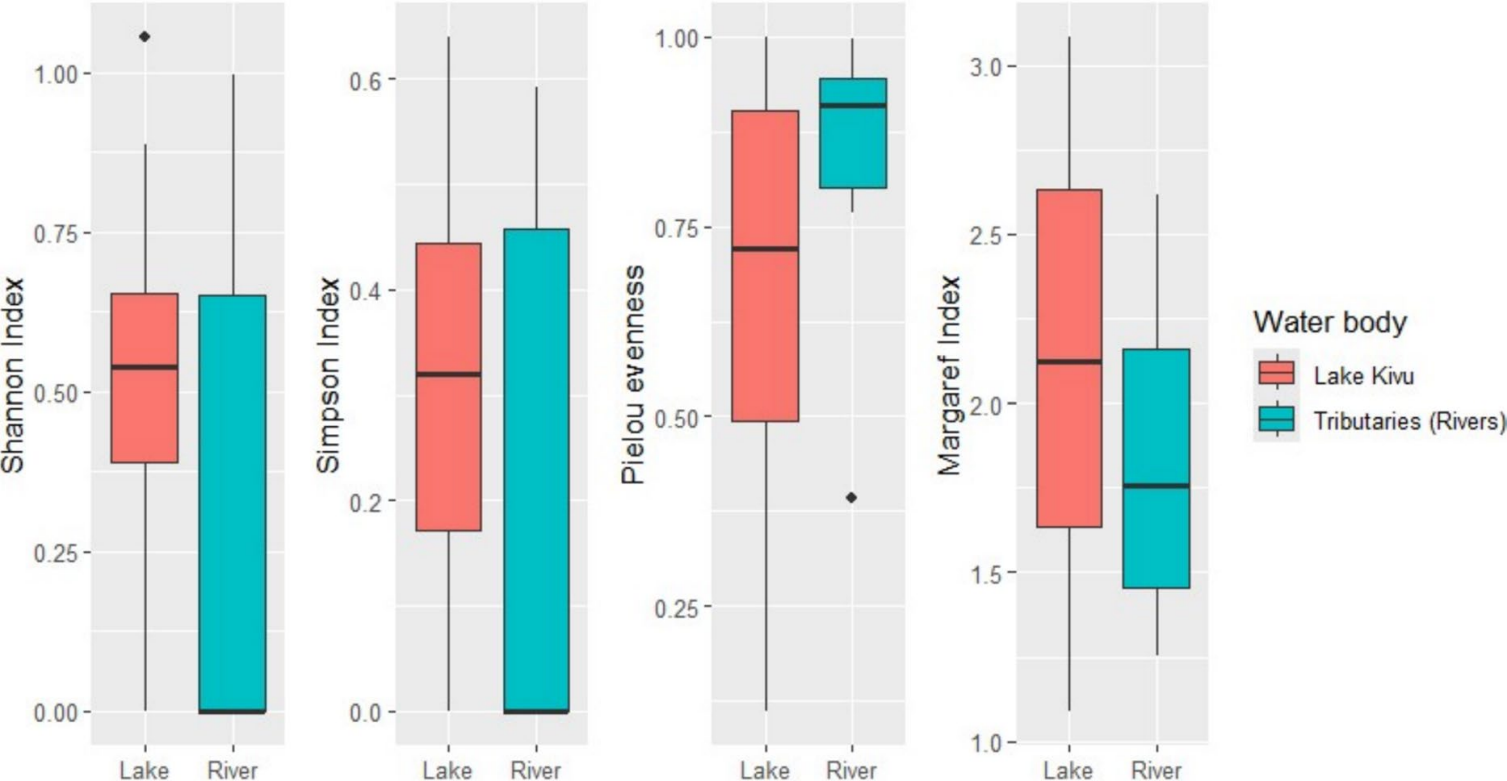
Diversity indices of molluscan species from Lake Kivu (*N* = 1894) and its tributaries (*N* = 878)

Diversity indices (Shannon, Simpson, Pielou, and Margalef) were also applied to molluscan species to assess variations in the community structure across the two water systems. The Wilcoxon test showed a significantly higher diversity in the lake than rivers as shown by the Shannon index (*p* = 0.02) and Simpson index (*p* = 0.03) (Fig. 4). However, the species’ evenness and richness did not significantly differ between the lake and rivers, as indicated by Pielou (*p* = 0.06) and Margalef index (*p* = 0.21; Fig. 4).

### Ecological health status of the water bodies

The values of ten metrics-MMIH applied to the three sections of the Lake (Table 2) range between 28 and 32 out of 50 possible score (Table 3). The northern and southern sections expressed the same index score (32) whereas the middle section had a slightly lower score (28). Thus, based on this index, these scores fall into the water quality category II (Northern and Southern), and category III (for middle section). This implies that the ecological status of Northern and Southern section is “good”, whereas the middle section reflected the conditions of “fair”. On the other hand, the rivers were classified in similar ways, applying MMIBI accordingly. Eleven metrics were used to score for macroinvertebrates (Table 3). The MMIBI values ranged from 29 to 31, whereby southern, middle, and northern parts scored 33, 31 and 29, respectively of the maximum score of 55 (Table 1). The MMIBI results showed that the Southern and Middle parts of catchment have moderate integrity class, where there is significant pollution levels and degradation. However, the northern part portends poor health status, with lower water quality characterized by the heavy pollution and degradation.

**Table 3:** Computed metrics from the applied multimetric indices, and their respective values. MMIH= Multimetric index for Lake Hawassa, RETC= Ratio of Ephemeroptera andTrichoptera to Chironomidae taxa, HBI= Hilsenhoff Biotic Index, MMIBI= Macroinvertebrate-based multimetric index for biotic integrity

**Lake Kivu**			
MMIH Metrics	Northern	Middle	Southern
%Ephemeroptera-Trichoptera taxa	0.71	0.61	0.23
%intolerant	12.64	1.59	2.93
%Trichoptera	0.28	0	0.11
%Diptera	4.31	1.35	0.93
%Chironomidae	4.31	1.35	0.93
% abundance of reference	29.46	34.41	36.11
% richness of reference	70.37	59.25	55.55
RETC	0.16	0.45	0.25
Ratio of intolerant to tolerant taxa	0.14	0.016	0.03
HBI	7.025	6.43	6.38
MMIH	32	28	32
**Lake Kivu tributaries**			
MMIBI Metrics	Northern	Middle	Southern
Number of Ephemeroptera taxa	172	308	199
Number ofTrichoptera taxa	51	161	56
Number ofHemiptera taxa	2	45	91
Shannon diversityindex	2.02	2.46193	2.33
% Hemiptera	0.28	2.7	4.94
%Trichoptera	7.28	9.67	3.04
%Tolerant taxa	42.71	42.36	46.63
%Dominant taxon	24.71	25.96	35.59
%scraper	51.71	24.09	15.38
%shredder	1.571	5.46	17.44
%filterers	8.71	16.34	3.48
MMIBI	**29**	**31**	**33**

**Fig. 5:**
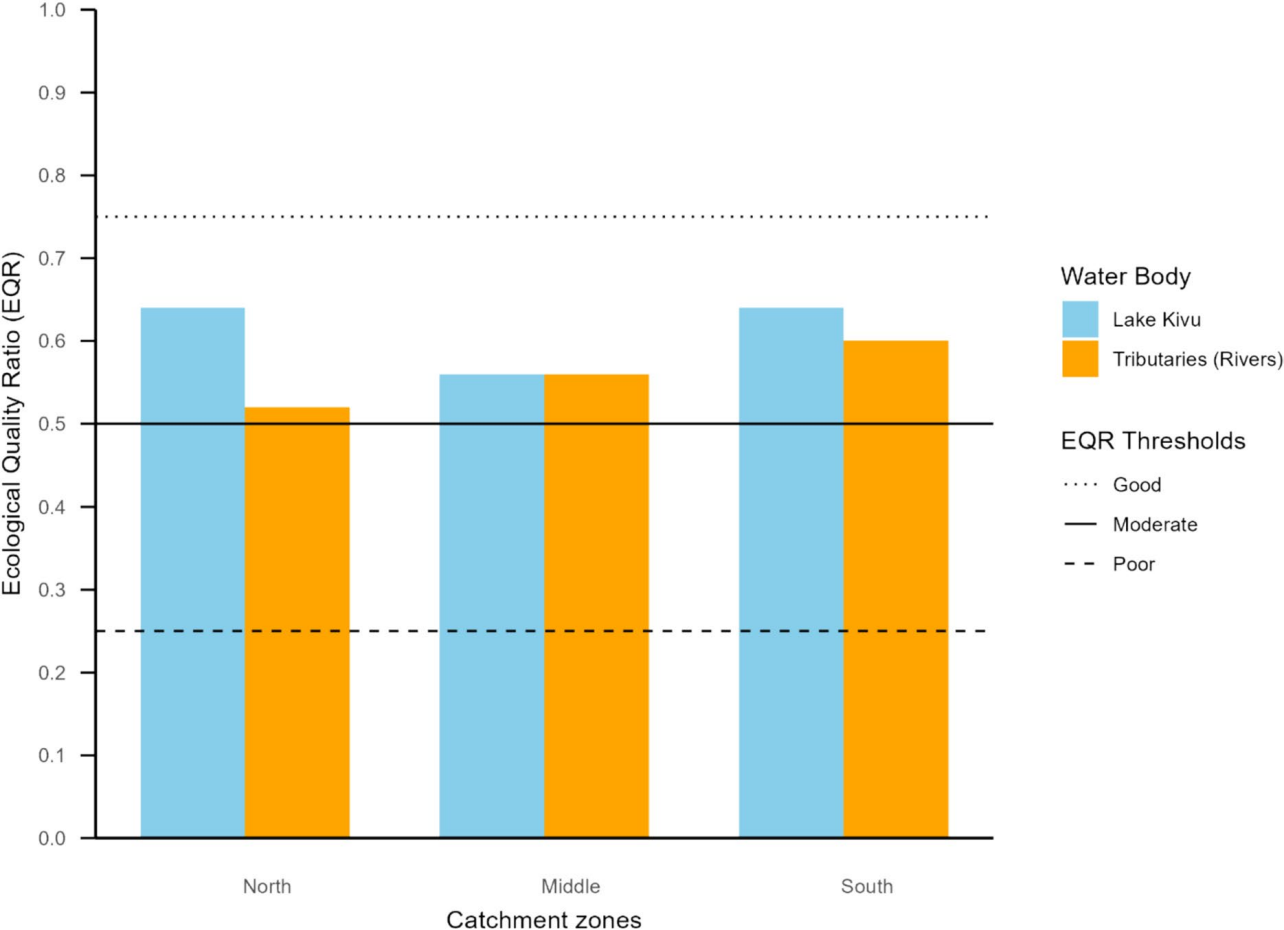
The Ecological Quality Ratio (EQR) for Lake Kivu and its tributaries across catchment zones, based on MMIH and MMIBI indices

The health status of the lake and rivers were compared using the Ecological Quality Ratio (Fig. 5). The lake expressed higher EQR in northern and southern sections than to their adjacent rivers with 0.64 to 0.52, and 0.64 to 0.6, respectively. However, the EQR did not differ in the middle section (Fig. 5). Although some difference occurred throughout the sections, the water quality of the lake and rivers is classified under moderate ecological health class (Fig. 5).

## Discussion

### Macroinvertebrate composition in Lake Kivu and its tributaries

Macroinvertebrate composition showed clear ecological differentiation between lake and tributary habitats, with molluscs being dominant in the lake, whereas insects dominated rivers. The lake was dominated by molluscs, particularly Bithyniidae and Planorbidae, which are well adapted to calm, nutrient-rich waters with stable substrates. Their dominance is consistent with previous findings that many gastropods favor lentic systems where biofilms and organic matter accumulate (Coelho et al., 2022; Jakubik et al., 2014). Planorbids, being air-breathing, tolerate low-oxygen conditions (Koopman et al., 2016), while gill-breathing bithyniids demonstrate metabolic stability across temperature ranges (Hahn, 2005) and effectively exploit nutrient-rich environments through filter-feeding (Trebitz et al., 2015). These traits make them suited to habitats dominating in Lake Kivu, where ion accumulation and carbonate sedimentation are prevalent (Hategekimana et al., 2020; Votava et al., 2017).

In contrast, the tributaries were dominated by insect orders such as Ephemeroptera and Odonata, taxa typically associated with fast-flowing, well-oxygenated environments. Members of Baetidae and Caenidae, for instance, exhibit strong swimming and clinging traits that help them persist in dynamic flow conditions (Clayton & Westbrook, 2008). Their abundance aligns with their role as indicators of sediment stress and flow regime in lotic systems (Akamagwuna et al., 2019; Principe et al., 2019). Odonates such as Coenagrionidae also showed affinity to lake habitats, likely benefiting from the structure and refuge offered by submerged vegetation (Villalobos-Jimenez et al., 2016; Verdonschot & Peeters, 2012), while others, like Gomphidae and Libellulidae, were more associated with flowing water, utilizing diverse substrates and prey availability (Calvão et al., 2020; Dijkstra et al., 2014).

Indicator taxa further emphasized the ecological distinctions between systems. In lakes, families such as Thiaridae, Bulinidae, Atyidae, Coenagrionidae, and Gomphidae showed strong associations with lentic conditions. This pattern reflects shared adaptations, including reliance on organic-rich sediments and submerged vegetation for feeding, reproduction, and refuge (Page et al., 2008; Villalobos-Jiménez et al., 2016; Remsburg, 2011). For instance, Atyidae and Thiaridae are detritivores that thrive in nutrient-rich benthic zones (Page et al., 2008; Bjelke and Herrmann 2005), while Coenagrionidae and Gomphidae depend on submerged structures for oviposition and predator avoidance (Villalobos-Jiménez et al., 2016; Remsburg, 2011). These shared traits enable them to exploit lacustrine environments effectively. In contrast, tributaries were characterized by families such as Hydropsychidae, Gyrinidae, and Protoneuridae, whose locomotion and respiration traits are well-suited to flowing water and associated prey availability (Raphahlelo et al., 2022; Reis et al., 2023; Wagner, 2000). These taxa not only reflect habitat structure but also provide strong bioassessment potential due to their trait-filtering responses along environmental gradients.

Species-level analysis of molluscs added further depth. Lake-dominant species such as *Melanoides tuberculata* and *Gabbiella humerosa* displayed high tolerance to pollution and hypoxia, with *M. tuberculata* capable of bioaccumulating heavy metals (Orabi & Khalifa, 2020). *G. humerosa*, including the endemic subspecies *G. humerosa kivuensis*, likely benefits from Lake Kivu’s chemical uniqueness, including elevated methane and carbon dioxide levels (Dusabe et al., 2024). In tributaries, *Radix natalensis* and *Pisidium kenianum* were more frequent, their presence associated with vegetated, oxygenated, shallow areas and fine sediment substrates (Loskotová et al., 2019; Soliman, 2008). These molluscs’ habitat preferences suggest functional diversity within the malacofauna, offering both bioindicator value and insight into spatial variation in environmental conditions across the basin.

### Diversity indices

The significantly higher diversity (Shannon and Dominance indices) observed in the rivers could be attributed to the dynamic flow, varied habitats along the course, and greater connectivity, supporting a wider range of species and ecological interactions. Indeed, the tributaries of Lake Kivu meander through diverse topographical features and landcovers including hills, forests, drylands, farmlands and residential areas (Muvundja et al., 2009; Wronski et al., 2015). This create a rich mosaic of ecological niches that supports a wide range of organisms. Lotic environments have been reported to have higher alpha diversity due to greater ecological heterogeneity and diverse sediment types (Allan & Castillo, 2007; Júnior et al., 2016). Along the river continuum, the flow regime, substrates and vegetation changes, creating a wide range of niches for different species (Grubaugh et al., 1997; Vannote et al., 1980). Moreover, the variation in habitats favors diverse feeding groups since tributaries receive a number of organic inputs such as nutrients and leaf litter (e.g. Doong et al., 2021; Masese et al., 2014b). On the contrary, lakes often exhibit limited colonization rate and less diversity (Johnson et al., 2004). In view of the family-level evenness and richness (Pielou and Margalef indices) between Lake and its tributaries, there was no significant difference. This implies that both lake Kivu and its tributaries exhibit a similar family diversity in relation to the entire population, and both have evenly distributed individuals per family.

Considering the molluscan species, the diversity indicated higher Shannon and Dominance indices in Lake Kivu than in tributaries. As discussed in the first paragraph of the discussion, a number of factors could have contributed to the higher diversity of molluscan species in the lake than rivers. Molluscs could have adapted to relatively stable hydrological conditions and water chemistry, providing efficient medium for growth and local differentiation (Bagalwa et al., 2024; Dusabe et al., 2024). Nonetheless, this varies based on the region and adaptability of the species. For instance, a study focusing on the effects of river–lake disconnection on freshwater molluscs found that disconnected lakes exhibited significantly lower species richness compared to connected systems (Zheng et al., 2024). This suggests that hydrological connectivity plays a crucial role in maintaining mollusc diversity in an ecosystem. In addition, suspended solids, substrate types and plant presence play an important role in shaping the molluscan communities (Lewin et al., 2023). The factors shaping Molluscan diversity extend beyond the fundamental habitat characteristics of water bodies, as previously mentioned, to encompass a broader range of environmental stressors. For instance, the decline in gastropods diversity in lakes Malawi and Tanganyika, reported by Van Bocxlaer et al., (2012), was attributed to surface water warming, increased runoff and eutrophication. Thus, specific characteristics of Lake Kivu such as desiccation events and volcanic activities (Wood & Scholz, 2017), would have also contributed to the present molluscan diversity status.

### Ecological health status of water bodies

The multimetric indices applied for assessment of the lake ecological status, classified Lake Kivu in “moderate” category (Fig. 4). This implies presence of water pollution and habitat degradation, but not yet at the critical levels (Wondmagegn & Mengistou, 2023). However, due to Lake Kivu’s large volume and exceptionally long residence time, ranging from 193 years to about 800 years in deep layers (GEF, 2020, Muvundja et al., 2023), the ecological effects of pollution may be delayed. Thus, the current moderate status may underestimate the cumulative and long-term effects of the past and ongoing pressures. Notably, activities such as factory operations, fisheries, and methane gas exploration (Olapade & Omitoyin, 2012; Balagizie et al., 2017), may already be affecting the lake’s water quality. This is supported by various forms of water pollution including organic and heavy metal contamination have been reported in different parts of the lake (Olapade & Omitoyin, 2012; Balagizie et al., 2017; Houbraken et al., 2017; Nsabimana et al., 2020; Nyiragatare et al., 2021). Moreover, population in the vicinity of Lake Kivu are engaging numerous potentially pollutant generating activities like farming, and small-scale industries/factories (Balagizie et al., 2017). The increasing pollution of the lake compromises the survival of the inhabiting organisms, especially at the littoral zone (Hyangya et al., 2021), which is an important zone for breeding, refugee and feeding of most aquatic organisms.

Both the northern and Southern sections of Lake Kivu exhibited relatively high Ecological Quality Ratios (EQR = 0.64), despite differences in the pollution of their adjacent tributaries. The northern section, located near Rubavu town, with highly polluted tributaries like Sebeya River, showed unexpectedly good ecological status. This discrepancy may be attributed to hydrodynamic factors such as increased water flow and wind-driven circulation, which could have led to dilution and dispersion of pollutants, thus reducing their impact on benthic communities. In fact, in-lake conditions such as habitat alterations and external pressures (e.g., land-use), have been reported to collectively homogenize macroinvertebrate community in lake (McGoff et al., 2013). In contrast, the southern section’s similarly high EQR corresponds more predictably with its relatively less polluted tributaries, indicating a better overall catchment condition. This observation is consistent with findings by Hyangya et al. (2021), who reported that the littoral zones of southern Lake Kivu exhibited water quality ranging from moderate to good. However, the middle section presented the lowest EQR among the other sections. This could plausibly be ascribed to the internal lake processes such as sediment resuspension. This lake internal pollution is recognized as a significant driver of aquatic ecosystem degradation, even in systems where external pollution is reduced (Wang & Jiang, 2016; Wei et al., 2021). It is often driven by wind and bioturbation, which release nutrients and pollutants from sediments (Li et al., 2018). These conditions are unfavorable for sensitive macroinvertebrate taxa and may favor tolerant species ultimately lowering multimetric index values.

The rivers from the northern side of the basin presented lower Ecological Quality Ratio (EQR) compared to the rest, owing to plausibly the structure and anthropogenic activities associated with these rivers. For instance, the River Sebeya flows through various landscapes accumulating pollutants from urban, agricultural and industrial areas. In fact, water quality analysis of Sebeya River revealed high levels of turbidity, total suspended solids, and chemical oxygen demand, particularly in settled areas (Uwacu & Akintande, 2019). Indeed, it experiences high levels of sedimentation, mainly in rainy seasons (Hahirwabasenga et al., 2024; Majoro et al., 2023). The combination of stressors colonized of the habitat dominated by the taxa tolerant to pollution and sedimentation. Moreover, the more polluted or damaged the habitat is, the more tolerant macroinvertebrate species will colonize the area (Adámek et al., 2010; Matlou et al., 2017). However, the Southern part of the basin shows a different trend since rivers run through rural areas with forested catchments. Indeed, rivers in the southern region of the basin are not highly affected by modernized agriculture and landslides, thus having favorable habitats. A study by Mupenzi et al. (2017) reported good quality of southern rivers owing to the forested catchment. In the present study, the middle side stood as the medium environment, between the northern and southern parts. They are relatively less urbanized than northern but with more human settlements than the Southern. Therefore, the EQR across the basin sides could possibly be attributed to anthropogenic activities, mainly the land-use and land cover of the catchment as the main source of pollution. In fact, EQR in riverine systems depend heavily on the surrounding landscape and associated activities (Wasson et al., 2010).

Tributaries of Lake Kivu were more polluted than the lake water, owing to plausibly their higher proximity to pollution than the lake. Although tributaries contribute much to pollution of the receiving lake (Marcarelli et al., 2019; Mooney et al., 2020), they are more susceptible to pollution than the receiving water bodies (Wieczorek et al., 2024). In fact, tributaries act as protective buffers against pollutants in lake–river systems, through degrading contaminant prior to reaching the lake (Wang et al., 2023). For instance, some rivers flow through urban (e.g.: Rivers Sebeya, Koko, Kilimbi, etc.), agricultural (e.g.: Rivers Sebeya, Ntaruko, Mugonero, etc.), and industrial areas (e.g.: Sebeya, Koko etc.), before reaching the lake. Thus, they accumulate domestic, agricultural runoffs, mining and the industrial wastes (Bagalwa et al., 2015; Hahirwabasenga et al., 2024; Olapade & Omitoyin, 2012; Wronski et al., 2015). Given their limited dilution capacity, the pollutants concentrate more compared to the larger volume in lake allowing dilution of pollutants and reducing the effect on macroinvertebrate community. In fact, dilution significantly reduces the pollutants in lakes (Shinohara et al., 2008; Welch et al., 2020). The dynamic of water pollution plays a crucial role in distribution and diversity of macroinvertebrates, hence perform their role as bioindicators. This takes place through eliminating the sensitive species and dominance of tolerant taxa in highly damaged sites. Some studies reported the same trend in water quality indication using macroinvertebrates in Lake Kivu basin (river/tributaries) (e.g.: Bagalwa et al., 2019; Baguma et al., 2024; Wronski et al., 2015), and water quality assessment was done, independently from tributaries (Hyangya et al., 2021, 2022). This research shed light on the linkage between lakes and tributaries, which would be backed up by regular monitoring to confirm this trend. Thus, it could inform effectively of the desired measures for sustainable conservation of this basin.

## Conclusion

The study investigated macroinvertebrate assemblages in the Lake Kivu basin by comparing the diversity and ecological status of Lake Kivu and its tributaries. The distinct composition of macroinvertebrate communities highlights the variation between these two interconnected systems. The presence and distribution of indicator taxa/species in these environments are potentially shaped by flow regime, substrate characteristics, and anthropogenic disturbances. As a result, these taxa demonstrate habitat specificity, strong ecological affinity, and bioindicator potential. Furthermore, the incorporation of molluscan species provided additional ecological insights, enhancing the understanding of macroinvertebrate assemblages in a deeper context. The study revealed that, while rivers supported general macroinvertebrate diversity, molluscan diversity was significantly higher in lakes. While the individual indices revealed various ecological status, from “fair” to “good” for lake and from “poor” to “moderate for rivers, the Ecological Quality Ratio (EQR) classified both systems within the moderate range (0.5–0.75). This provided valuable information for sustainable conservation strategies in the basin. Moreover, the investigated river–lake system provided a broader perspective on river–lake interactions.

Although the study assessed community composition and water quality in both Lake Kivu and its tributaries, it could not establish a causal influence of river on lake ecosystem. Thus, targeted research on spatial–temporal dynamics of these systems is needed to better understand the rivers’ influence on the lake. Higher taxonomic resolution for other groups such as insects is needed to deepen understanding of macroinvertebrate ecological patterns, relevant in future monitoring of the basin. While assessing the ecological status along the basin, the varied number of sites were considered per section. Although comparison was achieved, future studies would benefit from harmonized site clustering, particularly during sampling. Furthermore, the study recommends increased application of MMIs, and their validation for local efficiency to reduce potential errors associated with individual metrics in biomonitoring approaches. Sustainable land-use planning, riparian zone management, and pollution control practices in the catchment are also recommended to sustain the ecological integrity of this basin.

## Supplementary information

Below is the link to the electronic supplementary material.

Supplementary file (XLSX 539 KB)

## Data Availability

The data are available in supplementary materials.
